# Gasdermin-E Mediated Pyroptosis—A Novel Mechanism Regulating Migration, Invasion and Release of Inflammatory Cytokines in Rheumatoid Arthritis Fibroblast-like Synoviocytes

**DOI:** 10.3389/fcell.2021.810635

**Published:** 2022-02-14

**Authors:** Tao Wu, Xue-Pei Zhang, Qian Zhang, Yao-Yao Zou, Jian-Da Ma, Le-Feng Chen, Yao-Wei Zou, Ji-Meng Xue, Ruo-Fan Ma, Zhong Chen, Lie Dai

**Affiliations:** ^1^ Department of Rheumatology, Sun Yat-Sen Memorial Hospital, Sun Yat-Sen University, Guangzhou, China; ^2^ Department of Orthopedic Surgery, Sun Yat-Sen Memorial Hospital, Sun Yat-Sen University, Guangzhou, China

**Keywords:** fibroblast-like synoviocytes, pyroptosis, gasdermin E, migration, invasion, inflammatory cytokines, rheumatoid arthritis

## Abstract

Synovium fibroblast-like synoviocytes (FLSs) are important participants in the pathogenesis of synovitis and joint destruction in rheumatoid arthritis (RA). Pyroptosis is a pro-inflammatory and cell lytic programmed cell death mechanism mediated by gasdermin (GSDM) family proteins. In this study, we demonstrated the increased expression of GSDME and increased levels of GSDME-mediated pyroptosis in RA synovial tissues. *In vitro*, stimulation with TNF-α plus hypoxia mimicking the inflammatory and hypoxic environment in RA synovium induced GSDME-mediated pyroptosis in RA-FLSs in combination with the promotion of migration and invasion abilities and the release of inflammatory cytokines (IL-6, IL-8). Moreover, knockdown of GSDME significantly inhibited the proliferation rate, migration/invasion effects and cytokines released through the reduction of GSDME-mediated pyroptosis. The immunohistochemistry results showed that RA patients with high GSDME N-terminal (GSDME-NT) expression, which is the active form of GSDME, showed higher IL-6 expression in both lining and sublining layer of synovium than that in patients with low GSDME-NT expression, osteoarthritis and non-inflammatory orthopedic arthropathies. Our findings revealed a novel mechanism regulating cell proliferation, migration, invasion and inflammatory cytokines release during the process of GSDME mediated pyroptosis in RA.

## Introduction

Rheumatoid arthritis (RA) is an autoimmune disease characterized by chronic synovitis accompanied by excessive release of inflammatory cytokines and the destruction of bone and cartilage. Although the outcomes of RA patients have achieved considerable improvements due to treatments directed by “Treat to Target” strategy and other biological reagents, a significant proportion of patients fail to achieve clinical remission and experience radiographic joint damage progression (Lillegraven et al., 2012). It is urgently needed to identify novel therapeutic targets for RA.

Fibroblast-like synoviocytes (FLSs) located in the synovium have been identified as important contributors to the pathogenesis of RA due to their involvements in inflammation as well as cartilage and bone destruction (Bartok and Firestein, 2010). Increasing researches have been concentrated on the mechanisms underlying the abnormal proinflammatory responses and invasive behaviors of RA-FLSs and, hopefully, possible treatment strategies based on targeting RA-FLSs.

Pyroptosis is a form of programmed cell death featuring cell swelling and large bubbles blowing from the plasma membrane mediated by pore-forming gasdermin (GSDM) family proteins (Shi et al., 2017). Gasdermin is a family of proteins sharing a functionally important gasdermin N domain with pore-forming activity (Chadha et al., 2020). In the absence of activation signals, the gasdermin N domain and C domain are connected to each other and form an autoinhibitory structure (Ding et al., 2016). After cleavage between the N domain and C domain by caspases, the two parts are separated, and the N domain is disinhibited (Ding et al., 2016). The N terminal cleavages then bind to the cell membrane, oligomerize and form pores causing leakage of cell components, which is the pivotal and unique process of pyroptosis distinguished from other manners of cell death (Ding et al., 2016). Essentially, pyroptosis is a pro-inflammatory process because of its cell lytic activity, which mediates excessive release of inflammatory mediators and thus induces and aggravates inflammation (Shi et al., 2017). Based on this feature, pyroptosis might play a crucial role in the genesis and development of RA. A study has implicated the importance of GSDMD-mediated monocytes pyroptosis in RA pathology (Wu et al., 2020), and Ze-Qing Zhai’s latest article revealed a pathogenic role of GSDME in the pyroptosis of monocytes and macrophages in RA (Zhai et al., 2021). However, the occurrence of pyroptosis as well as its mechanism and effect in RA-FLSs remain unknown. In this study, we illuminated the role of GSDME-mediated FLSs pyroptosis in RA, providing a novel insight for the mechanisms of inflammatory cytokines secretion and aggressive cell behaviors in RA-FLSs.

## Material and Methods

### Reagent and Abs

Lipofectamine RNAiMAX reagent and opti-MEM medium were purchased from Thermo Fisher Scientific (United States). Small interfering RNAs (siRNAs) including GSDME (si-GSDME#1, stB0006132A; si-GSDME#2, stB0006132B; si-GSDME#3, stB0006132C) and control siRNAs (si-NC, siN0000001-1-10) were obtained from RiboBio (Guangzhou, China). The corresponding target sequences for GSDME silencing were si-GSDME#1, CCC​ACT​GCT​TCT​TTG​TAT​A; si-GSDME#2, CAA​GCA​GCT​GTT​TAT​GAC​A and si-GSDME#3, GGT​CCT​ATT​TGA​TGA​TGA​A. Primary antibodies including anti-human GSDME monoclonal ab (Abcam, ab215191), anti-human GSDME polyclonal ab (Proteintech, 13075-1-AP), anti-human-Thy1/CD90 ab (Abcam, ab181469), anti-human HIF-1α ab (Abcam, ab51608), anti-human Caspase-3 ab (Abcam, ab32351), anti-human IL-6 ab (Abcam ab9324) and anti-GAPDH ab (Cell Signaling Technology, #5174) were used in this study.

### Preparation of Human Synovium Tissue

Active RA patients fulfilled the 2010 American College of Rheumatology (ACR) /European League against Rheumatism (EULAR) classification criteria (Aletaha et al., 2010) and manifested with knee-joint swollen and pain were recruited from the Department of Rheumatology at Sun Yat-Sen Memorial Hospital between November 2020 and June 2021. RA synovium (*n* = 28) was obtained by Parker-Pearson needle biopsy. Control synovium was obtained from osteoarthritis (OA; *n* = 4), and non-inflammatory orthopedic arthropathies (Orth.A; *n* = 5) who received arthroplasty, or arthroscopy at the Department of Orthopedics as we previously described (Ma et al., 2014). The clinical characteristics of included participants were listed in Supplementary Table S1. The study was performed in accordance with the Declaration of Helsinki and approved by the Medical Ethics Committee of Sun Yat-Sen Memorial Hospital (SYSEC-2009-06 and SYSEC-KY-KS-012). All participants gave written informed consent.

### FLSs Culture and Intervention

FLSs were isolated using modified tissue culture method (Schumacher and Kulka, 1972) and culture as we previously described (Zhou et al., 2014). FLSs from passages 3 to 7 were used in this study. For TNF-α treatment, after serum starvation overnight, medium was replaced by serum-free DMEM containing recombinant TNF-α (R&D Systems, United States) with the indicated concentration. For hypoxia treatment, cells were cultured in a humidified Tri-gas incubator (1% O_2_, 5% CO_2_, and 94% N_2_) for the indicated time.

### Knockdown of GSDME Transcripts

RA-FLSs (10 (Ding et al., 2016) / well) were transfected with siRNAs(50 nM, 24 h)using Lipofectamine RNAiMAX reagent and Opti-MEM medium following the manufacturer’s instructions.

### Quantitative Real-Time PCR

Total RNA was prepared from cells using RNAiso Reagent (Takara, Japan). Complementary DNA (cDNA) samples were synthesized with reverse-transcription kit (Takara, Japan). Primers used for amplification of the cDNA were as follows (forward, reverse): GSDMA, 5′-CTG​ACA​CCA​CTT​GAC​AGC​CT-3′ and 5′-TTC​AGG​AAT​GGG​TGG​TCT​GC-3’; GSDMB, 5′-TCT​CAG​GGC​CAT​CTC​AGC​TA-3′ and 5′-AGC​ACC​ATC​CTT​CTT​CAT​CGT-3; GSDMC, 5′-GGA​AGC​AAA​GAC​CTG​ACA​CC-3′ and 5′- GGA​ACG​GTC​CTG​TCA​CAA​CA-3; GSDMD, 5′-CTG​CTC​CAT​GAG​AGG​CAC​C-3′ and 5′-AGG​ACG​TCC​AAG​TCA​GAG​TCA-3; GSDME, 5′-TGA​TGG​TGA​CCT​GAT​TGC​AG-3′ and 5′- CGA​CCA​CTG​GAC​TCG​GAA​AT-3. qPCR was performed using QuantiTeckTM SYBR Green PCR kit (Takara, Japan). The reactions were initiated with denaturation of cDNA templates at 95°C for 30 s, 95°C for 5 s and 60°C for 30 s and amplification for 40 cycles on a Roche LightCycler480 sequence detector system (Roche, Switzerland).

### EdU Proliferation Assay

The EdU staining was conducted using a BeyoClick EdU Cell Proliferation Assay Kit (Beyotime, China) according to the manufacturer’s protocol. Images were captured using an Olympus IX73 microscope and the number of cells was manually counted.

### Immunohistochemistry & Immunofluorescence Staining

IHC staining was carried out on the qualified synovium as we previously described (Ma et al., 2014). Antigens were retrieved by boiling in EDTA (pH 9.0) for 3 min at 120°C in a pressure cooker. Sections were then washed, blocked, and incubated with rabbit anti-GSDME ab (Proteintech, 1:100) or mouse anti-IL-6 ab (Abcam, 1.5 ug/ml) overnight at 4°C. After washing, the sections were incubated with EnVision Mouse or Rabbit conjugate (Dako Corporation, United States) for 30 min at 37°C. The color reaction was completed with the DAB-positive substrate and hematoxylin. Concentration-matched normal rabbit IgG (Cell Signaling Technology, #2729) and normal mouse IgG (Santa Cruz, sc-2025) were used as a negative control. The mean IL-6 expression in the synovium in each field was valuated under a 400× optical microscope using the scoring system (Huang et al., 2018) multiplied the intensity of the scoring (0, = absent, 1 = weak, 2 = moderate, 3 = strong) and the percentage of positive cells (0 = 0%, 1 = 1–25%, 2 = 26–50%, 3 = 51–75%, 4 = 76–100%). The mean score from five different fields was determined as the finial IHC score.

For IF staining, sections were incubated with rabbit anti-GSDME ab (Proteintech, 1:100) and mouse anti-CD90 Ab (Abcam, 1:100) overnight at 4°C and then incubated with Alexa Fluor 594 conjugated anti-rabbit IgG (Cell Signaling Technology, #8889, 1:800) and Alexa Fluor 488 conjugated anti-mouse IgG (Cell Signaling Technology, #4408, 1:800) for 30 min at 37°C. After staining with DAPI slides were observed using a laser confocal microscope (LSM 510; Carl Zeiss, Germany).

### Flow Cytometry Analysis for Cell Death

RA-FLSs (1.5 × 10^5^ / well) treated as indicated were collected and stained with Annexin V-FITC/propidium iodide (PI) Apoptosis Detection kit (Elabscience, China). Cell death analysis was performed on the BD FACS Caliber flow cytometer.

### PI Staining and Microscopy Imaging

RA-FLSs were treated as indicated in 24-well plates and incubated with 1 μg/ml PI (Solarbio, China) for 15 min at 37°C. Brightfield video and static brightfield / PI channel images of pyroptotic cells were captured using an Olympus IX73 microscope.

### Lactate Dehydrogenase Assay

RA-FLSs (10 (Chadha et al., 2020) /well) were seeded into 96-well culture plates and treated as indicated. The supernatant was collected and determined for LDH activity using LDH Assay Kit (Beyotime, China) according to the manufacturer’s instruction.

### Western Blot Analysis

RA-FLSs were collected and the total protein was extracted using Column Tissue & Cell Protein Extraction Kit (Epizyme, China) following the manufacturer’s protocol. Concentrations of extracted protein were determined using the Pierce BCA assay kit (Thermo Fisher Scientific, United States). Expression of proteins were analyzed using western blot as we previously described (Ma et al., 2019a).

### Cell Migration and Invasion Assays

#### Wound Healing Assay

RA-FLSs (10 (Ding et al., 2016)/well) were seeded in six-well plates and treated as indicated. Wound lines were made by scratching the cellular monolayer with sterile 200 µL pipette tips. After 48 h of incubation in serum-free medium, cell migration was measured by calculating that healing area beyond reference line at 0 h using ImageJ software.

#### Transwell Migration and Invasion Assay

Migration and invasion assay were performed using Transwell chambers as we described previously (Ma et al., 2019b). For migration assay, 2 × 10^4^ RA-FLSs were resuspended by serum-free DMEM, placed in the upper chamber, and allowed to migrate for 12 h. For invasion assay, the upper chamber was pre-coated with Matrigel (1:5) (BD Biosciences, United States) and the same amount of FLSs were resuspended by serum-free DMEM, placed in the upper chamber allowed to invade for 24 h. 10% FBS was placed in the lower chamber as a chemoattractant in both migration and invasion assay. After removal of cells remained on the upper surface of the membrane, the cells on the lower surface were stained with crystal violet staining solution (Solarbio, China) manually counted. Data were presented as the mean number of cells in five randomly captured 100× fields.

### Cytokines Assays

Supernatants of cells with the indicated treatments were collected and centrifuged (12,000 rpm, 5 min), and secretion of cytokines was analyzed by a Protein Profiler Human Cytokine Array Kit (ARY005B, R&D Systems, United States) following the manufacturer’s protocol. Results were quantified using ImageJ software. Secretion levels of IL-6, IL-8 were also detected using corresponding ELISA kits (Neobioscience, China) following the manufacturer’s protocol.

### Statistical Analyses

Statistical analyses were performed on SPSS 25.0 statistical software (SPSS, United States). Data were presented as mean ± standard error of mean (s.e.m). Comparisons between two groups were analyzed by two-tailed Student’s t test. Multiple groups of samples were analyzed by one-way ANOVA, and pairwise comparisons were adjusted by the Bonferroni method. Differences were considered statistically significant with P values < 0.05.

## Results

### GSDME Expression in Synovium and FLSs

Using the data exploration website developed by Lewis, M. J., et al. (https://peac.hpc.qmul.ac.uk/) (Lewis et al., 2019), GSDME (DFNA5) gene signatures were dissected across different synovial compartments in the Pathobiology of Early Arthritis Cohort (PEAC) and integrated with deep phenotypic profiling. GSDME expression was found in synovial tissue, and this gene was highly expressed in fibroid subtypes (Supplementary Figure S1A). Furthermore, weighted correlation network analysis (WGCNA) showed that GSDME was annotated against THY-1(+) fibroblast cell population (Supplementary Figure S1B), which was found to be remarkably expanded in RA synovium compared to control synovium and correlated with severe and persistent inflammation in RA (Croft et al., 2019; Wei et al., 2020).

**FIGURE 1:**
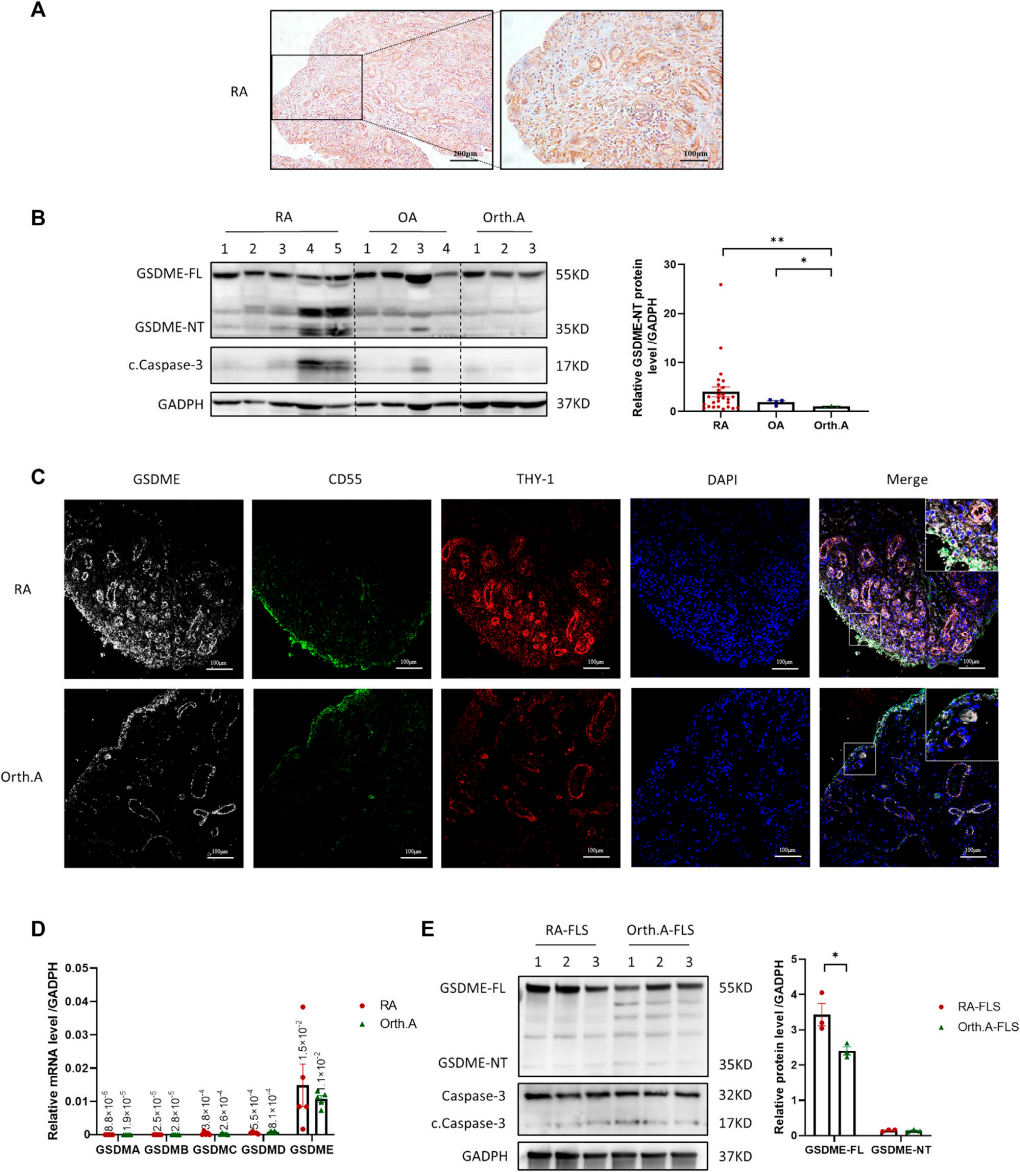
Expression of GSDME in synovium tissue and RA-FLSs. **(A)** Representative IHC staining of GSDME in synovium from RA patients (scale bar = 200 / 100 μm). **(B)** Western blot of cleaved caspase-3, GSDME and N-terminal cleavage expression in lysate of synovium tissues from RA (*n* = 27), OA (*n* = 4) and Orth.A (*n* = 3) patients. **(C)** Representative immunofluorescence staining of GSDME (Gery), CD55 (Green), THY-1 (Red) and DAPI (Blue) in synovium of RA and Orth. A patients (scale bar = 100 μm). **(D)** GSDMA-E mRNA expression in FLSs from RA (*n* = 5) and Orth. A (*n* = 5) patients evaluated by qPCR. **(E)** GSDME-FL, GSDME-NT, caspase-3 and cleaved caspase-3 protein expression in FLSs from RA patients (*n* = 3) and Orth.A patients (*n* = 3) evaluated by western blot. Data are shown as mean ± s.e.m. *p < 0.05, **p < 0.01 using one-way ANOVA.

To confirm the expression of GSDME in RA synovial tissue, 5 active RA patients with distinct knee joint pain were recruited, biopsied, and IHC staining was then conducted on these samples. GSDME full-length protein (GSDME-FL) expression was detected in both the lining and sublining layers (Figure 1A). As the key step of pyroptosis, GSDME-FL is cut into an N-terminal fragment (GSDME-NT) with pore-forming activity and a C-terminal fragment by the activated cleaved caspase-3 and then cell pyroptosis is induced (Ding et al., 2016; Wang et al., 2017; Jiang et al., 2020). To explore the existence of GSDME-mediated pyroptosis in synovium from RA patients and “less inflamed” disease controls, western blot analysis were performed for the detection of activated GSDME-NT in synovial tissue extracts from RA, OA, and Orth.A patients. As shown, GSDME-NT and GSDME-FL expression was significantly increased in synovium extracts from RA patients compared to those from Orth.A patients, but statistic difference was not found in either GSDME-FL or GSDME-NT levels between OA synovium and Orth.A synovium (Figure 1B, Supplementary Figure S2). Immunofluorescence staining of RA and Orth.A synovium also revealed colocalization of GSDME with CD55 in the lining area and THY-1 FLSs in the sublining area, suggesting a link between FLSs and GSDME-mediated pyroptosis in RA synovium as detected above (Figures 1C, Supplementary Figure S3). Furthermore, in FLSs cultured *in vitro*, GSDME mRNA, not GSDMA, GSDMB, GSDMC, or GSDMD, was the most abundantly transcribed, but no statistically significance was found between the mRNA levels of RA-FLSs and Orth. A-FLSs (Figure 1D). Protein levels of GSDME-FL and GSDME-NT were also detected by western blot. Results showed that GSDME-FL protein expression was increased in RA-FLSs compared to Orth.A-FLSs, but GSDME-NT expression showed no difference, possibly due to the absence of disease microenvironment *in vitro* (Figure 1E).

### Inflammatory and Hypoxic Microenvironment Triggers GSDME-Mediated RA-FLSs Pyroptosis

**FIGURE 2:**
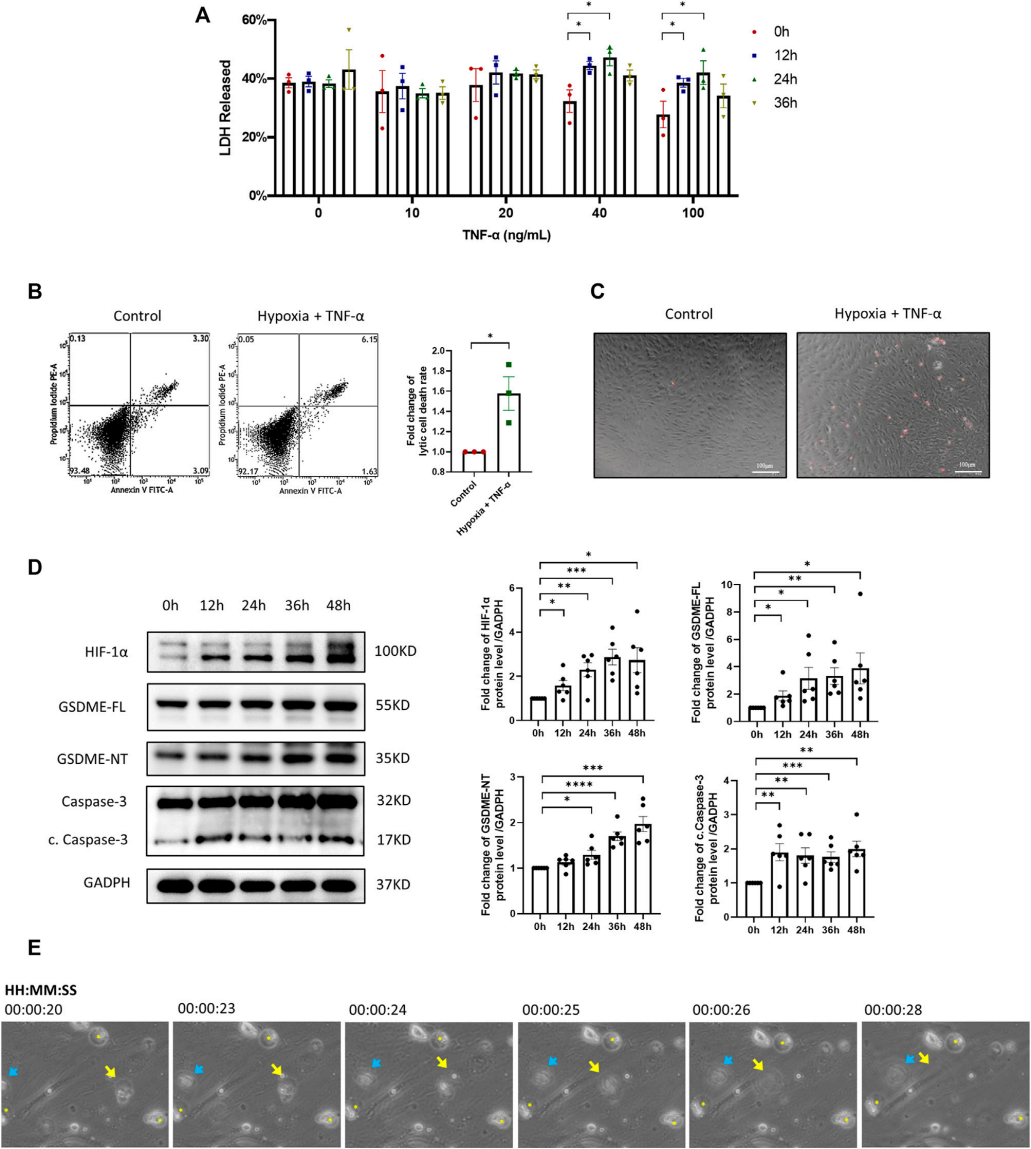
Inflammatory and hypoxic microenvironment triggers GSDME-mediated RA-FLSs pyroptosis. **(A)** LDH released by RA-FLSs treated with indicated concentrations of TNF-α under hypoxia conditions for 0-36 h (*n* = 3). **(B)** Flow cytometry analysis (*n* = 3) of Propidium Iodide (PI) and Annexin V stained cells with or without TNF-α + Hypoxia treatment (40 ng/ml, 36 h). **(C)** Representative phase-contrast images with PI staining by different treatment (scale bar = 100 μm). **(D)** Western blot analysis of caspase-3, cleaved caspase-3, GSDME and N-terminal cleavage of RA-FLSs treated with 40 ng/ml TNF-α under hypoxia conditions for 0-48 h (*n* = 6). HIF-1α serves as a marker of effective hypoxic stimulus, GADPH serves as a loading control. **(E)** Video captures of RA-FLSs under TNF-α + Hypoxia treatment (40 ng/ml, 36 h). Blue arrow and yellow arrow point out two bursting cells. Yellow asterisks designate the swelling cells. Data are shown as mean ± s.e.m and results are representative of three independent experiments. *p < 0.05, **p < 0.01, ***p < 0.001, ****p < 0.0001 using one-way ANOVA.

It has been well recognized that the inflammatory and hypoxic microenvironment in RA synovium facilitates the abnormal activation of RA FLSs. To mimic the microenvironment in RA synovium, RA-FLSs were treated with TNF-α and exposed to hypoxia (1% O_2_). The LDH release test (Figure 2A) showed that treatment with 40 ng/ml TNF-α plus a 36-h hypoxia exposure represented the optimal trigger for lytic cell death of RA-FLSs and used in the subsequent experiments. The proportion of lytic cell death determined by flow cytometry analysis and microscopic PI staining was also significantly increased after treatment with TNF-α plus hypoxia (Figures 2B,C). Moreover, characteristic swelling of cells, formation of large bubbles from plasma membrane, and eventually cell rupture of RA-FLSs were observed after treatment with TNF-α plus hypoxia, establishing the characteristic morphology of the pyroptosis process (Figure 2E). Consistent with the abovementioned evidences, increased expression of GSDME-NT in RA FLSs treated with TNF-α plus hypoxia was detected by western blot (Figure 2D), further suggesting that TNF-α plus hypoxia triggers GSDME-mediated pyroptosis in RA-FLSs. Moreover, increases in HIF-1α, GSDME-FL and cleaved caspase-3 expression were also observed after TNF-α plus hypoxia treatment (Figure 2D).

### Downregulation of GSDME Alleviates TNF-α Plus Hypoxia-Induced RA-FLSs Pyroptosis

**FIGURE 3:**
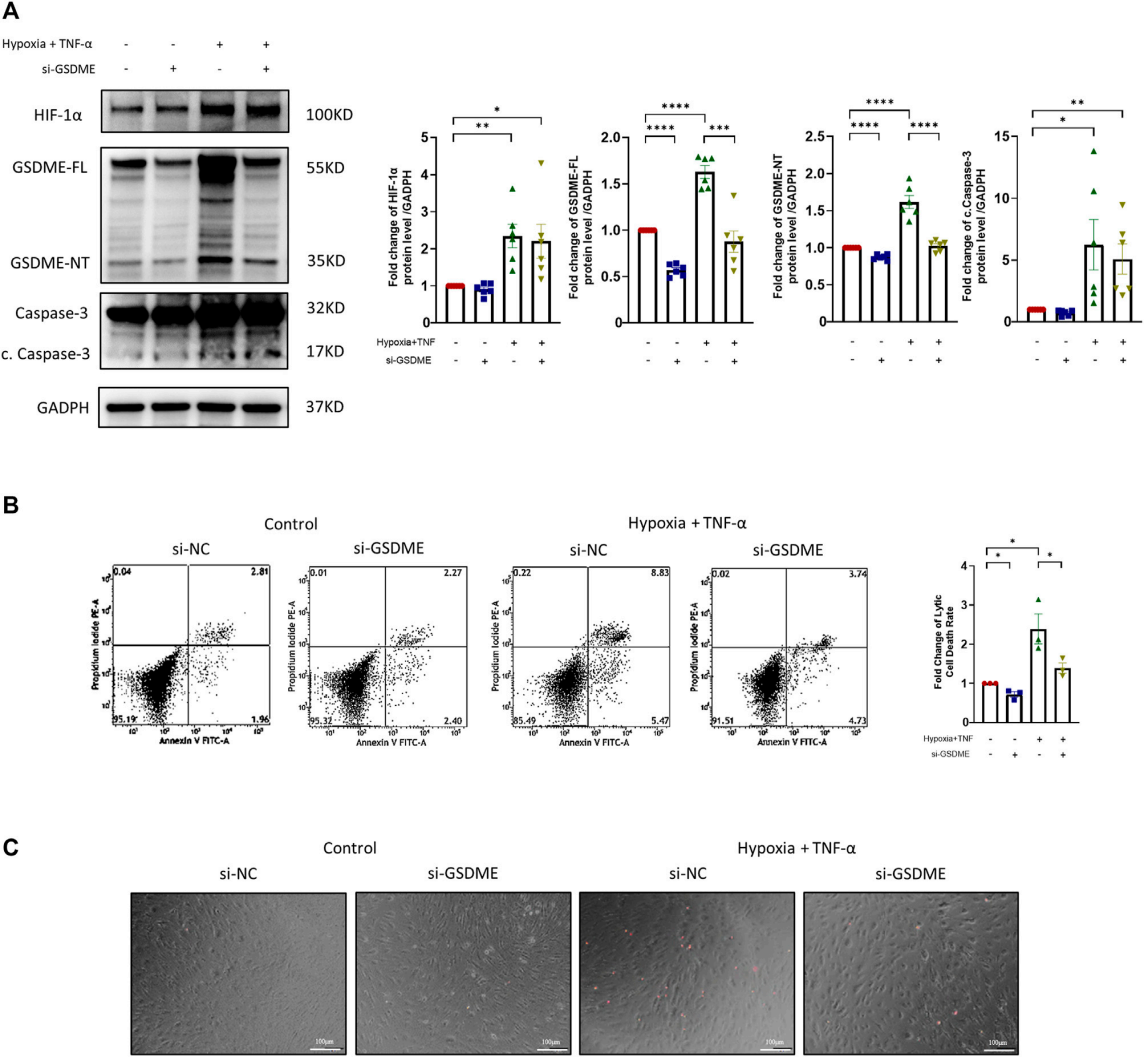
Suppression of RA-FLSs pyroptosis by downregulation of GSDME. **(A)** Western blot analysis of casepase-3, cleaved caspase-3, GSDME and HIF-1α of RA-FLSs treated with TNF-α + hypoxia treatment (40 ng/ml, 36 h). GSDME siRNA or control siRNA was transfected for 24 h before treatment (*n* = 6). GADPH serves as a loading control. **(B)** Flow cytometry analysis (*n* = 3) and **(C)** representative phase-contrast images with PI staining of RA-FLSs treated with or without TNF-α + hypoxia (40 ng/ml, 36 h) after 24 h of transfection with GSDME siRNA (si-GSDME) or control siRNA (si-NC). Data are shown as mean ± s. e.m and results are representative of three independent experiments. *p < 0.05, **p < 0.01, ***p < 0.001, ****p < 0.0001 using one-way ANOVA.

The silencing efficiency of GSDME siRNA was confirmed by qRT-PCR and western blot (Supplementary Figures S3A,B). After TNF-α plus hypoxia treatment, the expression levels of HIF-1α, cleaved caspase-3, GSDME-FL and GSDME-NT were significantly increased (Figure 3A). However, the protein expression levels of GSDME-FL and GSDME-NT were decreased by GSDME siRNA transfection in both the control group and treatment group. Meanwhile, knockdown of GSDME lowered the rate of lytic cell death in both the control and treatment groups. (Figures 3B,C). The results indicated that downregulation of GSDME mitigated RA FLSs pyroptosis induced by TNF-α plus hypoxia.

### Suppression of RA-FLS Proliferation, Migration, and Invasion Through GSDME Knockdown

**FIGURE 4:**
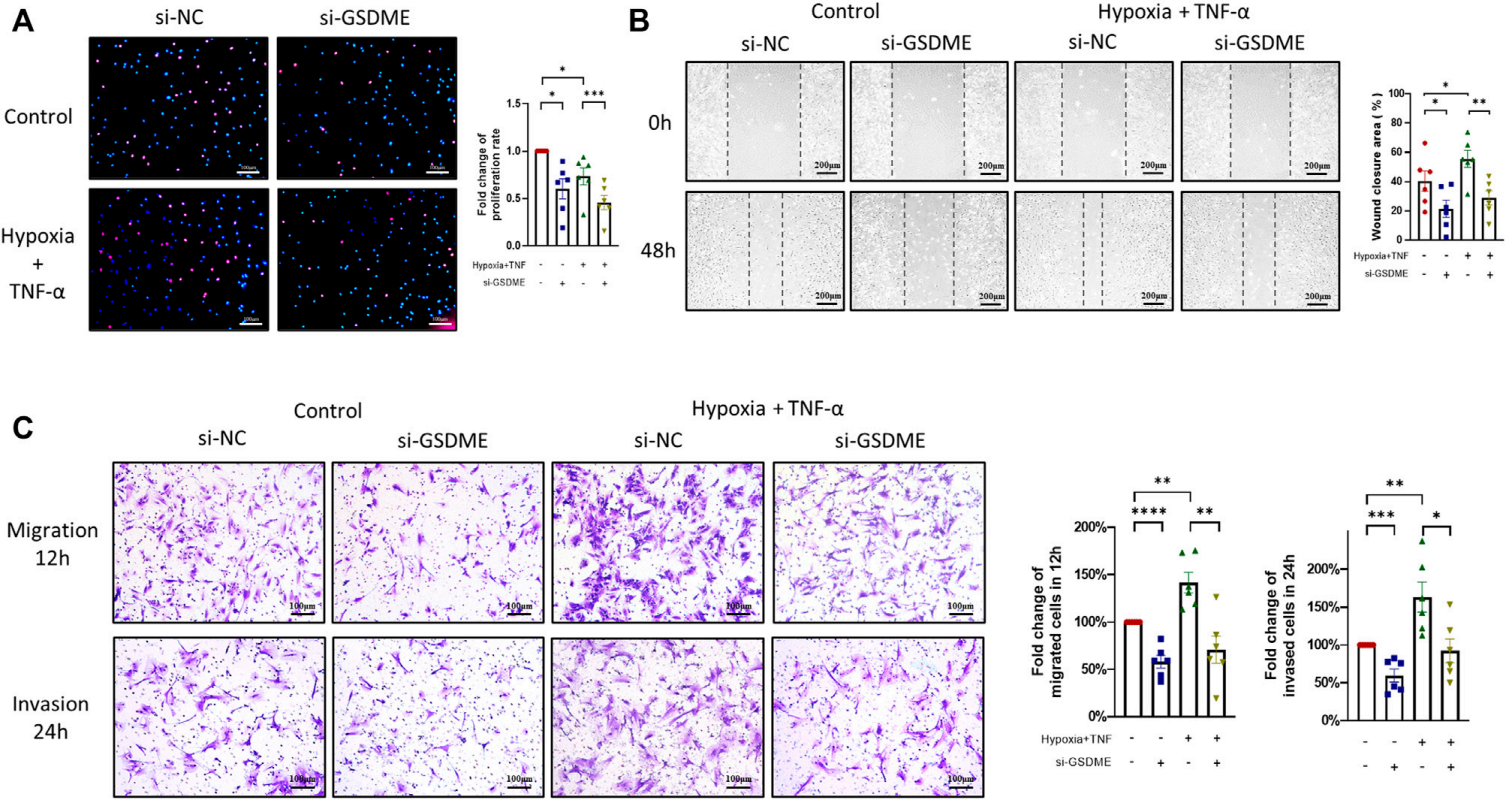
Suppression of proliferation, migration and invasion of RA-FLSs through inhibition of GSDME-mediated pyroptosis. **(A)** Decrease in RA-FLSs proliferation by knockdown of GSDME measured by EdU experiment (*n* = 6, scale bar = 100 μm). **(B,C)** Inhibition of RA-FLSs migration and invasion activities by GSDME knockdown. After 24 h of transfection with GSDME siRNA (si-GSDME) or control siRNA (si-NC), RA-FLSs migration was determined by wound healing assay (B, *n* = 6, scale bar = 200 μm) and transwell chamber assay (C, *n* = 6, scale bar = 100 μm), invasion was determined for by matrigel-coated transwell assay (C, *n* = 6, scale bar = 100 μm). Data are shown as mean ± s.e.m and results are representative of three independent experiments. *p < 0.05, **p < 0.01, ***p < 0.001, ****p < 0.0001 using one-way ANOVA.

Excessive proliferation, abnormal migration and invasion are the basic pathologic characteristics of RA FLSs. Results of EDU assay showed that both TNF-α+hypoxia treatment and GSDME knockdown restrained the proliferation of RA-FLSs (Figure 4A). On the other hand, silencing GSDME suppressed RA-FLSs migration and invasion abilities stimulated by TNF-α+hypoxia treatment (Figures 4B,C). Taken together, these results suggested a pivotal role of GSDME-mediated pyroptosis in the phenotypic transformation of RA FLSs.

### Attenuation of Inflammatory Cytokines and Chemokines Release by Inhibition of GSDME-Mediated RA-FLSs Pyroptosis

**FIGURE 5:**
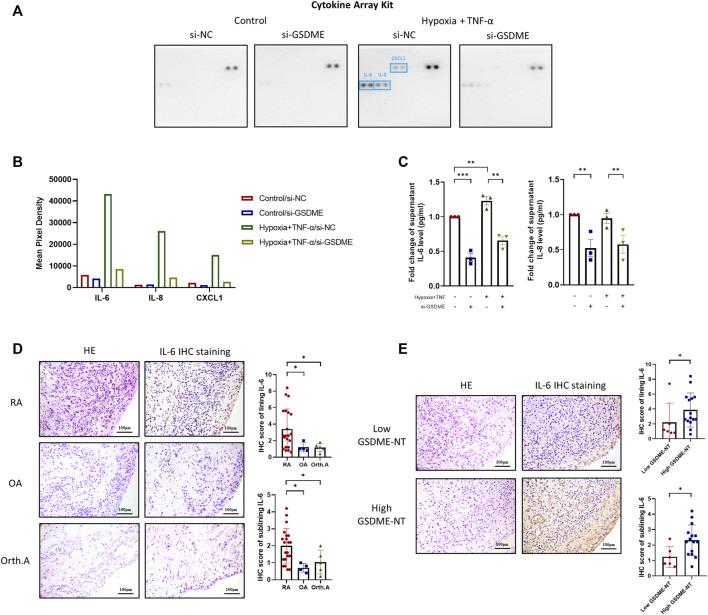
Alleviation of IL-6 and other inflammatory cytokines release by downregulation of GSDME-mediated RA-FLSs pyroptosis. **(A,C)** After siRNA transfection and TNF-α + hypoxia treatment, cell culture supernatant was collected and the levels of cytokines and chemokines were measured using Proteome Profiler Human Cytokine Array Kit from R&D systems **(A,B)**. Supernatant levels of IL-6, IL-8 were further verified by ELISA (n = 3) **(C)**. **(D,E)** HE and IHC staining for IL-6 of synovium tissues from RA (*n* = 21), OA (*n* = 4) and Orth.A (*n* = 5) patients (scale bar = 100 μm). RA synovium tissues were futher divide into low GSDME-NT level (*n* = 6) and high GSDME-NT level (*n* = 15) groups based on matched GSDME-NT level determined by western blotting. Data are shown as mean ± s. e.m and results are representative of three independent experiments. *p < 0.05, **p < 0.01, ***p < 0.001 using one-way ANOVA.

Pyroptosis is characterized by cell rupture accompanied by release of a great quantity of cell components, including proinflammatory cytokines and chemokines, which are key regulators of cell behaviors in RA FLSs. To further investigate the underlying mechanisms, supernatants of RA FLSs after TNF-α plus hypoxia treatment were collected and detected by a Proteome Profiler Array Kit to identify the key cytokines and chemokine responding to GSDME knockdown. After TNF-α+hypoxia stimulation, supernatant IL-6, IL-8 and CXCL1 level were remarkedly increased. Reversely, GSDME knockdown attenuated IL-6, IL-8 and CXCL1 release in response to TNF-α and hypoxia treatment. (Figures 5A,B). The regulation of IL-6, IL-8 and release by GSDME knockdown was further validated by corresponding ELISA assay (Figure 5C).

### Association Between GSDME-Mediated Pyroptosis and IL-6 Levels in RA Synovium

Based on the aforementioned results, suppression of GSDME-mediated RA-FLSs pyroptosis dramatically decreased the levels of IL-6 released *in vitro*, implicating a novel mechanism by which IL-6 and other cytokines may be excessively released under inflammative and hypoxic circumstance such as RA. To verify this hypothesis in synovial samples, twenty-one RA, four OA and five Orth. A patients with qualified synovium were subjected to IHC analysis of the synovial IL-6 expression. IL-6 expression was observed in both the lining and sublining layers of the synovium (Figure 5D). The median IHC scores of both lining and sublining IL-6 expression were significantly higher in RA patients than that in OA [Lining: 2.6 (1.2, 5.5) *vs*. 1.0 (0.9, 1.8), *p* = 0.049; Sublining: 2.2 (1.2, 2.6) *vs*. 0.7 (0.5, 1.0), *p* = 0.011], or Orth. A patients [Lining: 2.6 (1.2, 5.5) *vs*. 1.2 (0.7, 1.6), *p* = 0.037; Sublining: 2.2 (1.2, 2.6) *vs*. 1.0 (0.4, 1.7), *p* = 0.047]. RA patients were further classified into a high GSDME-NT expression group (*n* = 15) and a low GSDME-NT expression group (*n* = 6) according to the expression level of GSDME-NT in Orth.A determined by western blot analysis of synovial tissue extracts. Patients with expression levels greater than that noted in Orth.A were classified as high GSDME-NT expression; otherwise, patients were considered to have low GSDME-NT expression. RA patients with high GSDME-NT expression possess higher IL-6 IHC scores than those with low GSDME-NT expression [Lining: 2.8 (2.4, 5.6) *vs*. 1.1 (0.8, 3.4), *p* = 0.047; Sublining: 2.2 (1.4, 2.8) *vs*. 1.0 (0.8, 1.8), *p* = 0.029], suggesting that GSDME-mediated pyroptosis can obviously affect the synovial IL-6 level (Figure 5E).

## Discussion

In the present study, for the first time, we reported the occurrence of GSDME-mediated pyroptosis in RA-FLSs under the inflammatory and hypoxic synovial microenvironments. Moreover, our results indicated that GSDME-mediated pyroptosis contributes to the proliferation, migration, invasion, and release of inflammatory cytokines in RA-FLSs, further providing a novel direction for RA pathology research and treatment development.

As a basic cell biological process, miscellaneous forms of cell death have been studied in RA-FLSs, and the most well-known is apoptosis—a non-proinflammatory programmed cell death pattern. RA-FLSs are resistant to apoptosis and present with excessive proliferation and aggressive cell behaviors, including enhanced secretion of inflammatory and chemotactic cytokines and increased migrative and invasive ability. Induction of apoptosis in RA-FLSs through certain therapies is therapeutically beneficial in RA (Pope, 2002; Smith et al., 2010). Differ from apoptosis, pyroptosis exhibits a pro-inflammatory peculiarity. Due to the formation of gasdermin pores on the plasma membrane, cells undergo osmolysis, and excessive cellular contents, including inflammatory cytokines and danger-associated molecular patterns (DAMPs), are released into extracellular compartment (Shi et al., 2017; Chadha et al., 2020), thereby inducing and exaggerating inflammation. Pyroptosis has been implicated in cancer biology and infectious disease (Wang et al., 2017; Sarhan et al., 2018; Jiang et al., 2020; Karki et al., 2021). Moreover, several researches in osteoarthritis indicated that GSDMD-mediated FLSs pyroptosis participates in HIF-1α-induced synovial fibrosis in knee osteoarthritis (Zhao et al., 2018; Zhang et al., 2019). A recent study also showed that GSDMD-mediated monocytes pyroptosis was involved in persistent inflammatory cytokine release in RA pathology (Wu et al., 2020). The latest research ultimately studied the pathogenic role of GSDME in monocytes, macrophages and animal models of RA, suggesting that GSDME might be a potential therapeutic target for RA (Zhai et al., 2021). However, involvement of GSDME-mediated pyroptosis in the function of RA-FLSs has not yet been reported.

Interestingly, we found that both full-length GSDME and GSDNE-N terminal cleavage was detected in RA-FLSs in synovial tissue *in situ* and cultured *in vitro*, constituting the prerequisite of pyroptosis. In addition, by mimicking the particular microenvironment in which RA-FLSs are situated inflaming and hypoxic circumstances due to long-term and severe synovitis (Fearon et al., 2016), we observed that a larger proportion of RA-FLSs undergo lytic cell death along with activated GSDME-NT expression and typical morphological features, strengthening the occurrence of GSDME-mediated RA FLSs pyroptosis in inflammatory and hypoxic microenvironments.

**FIGURE 6:**
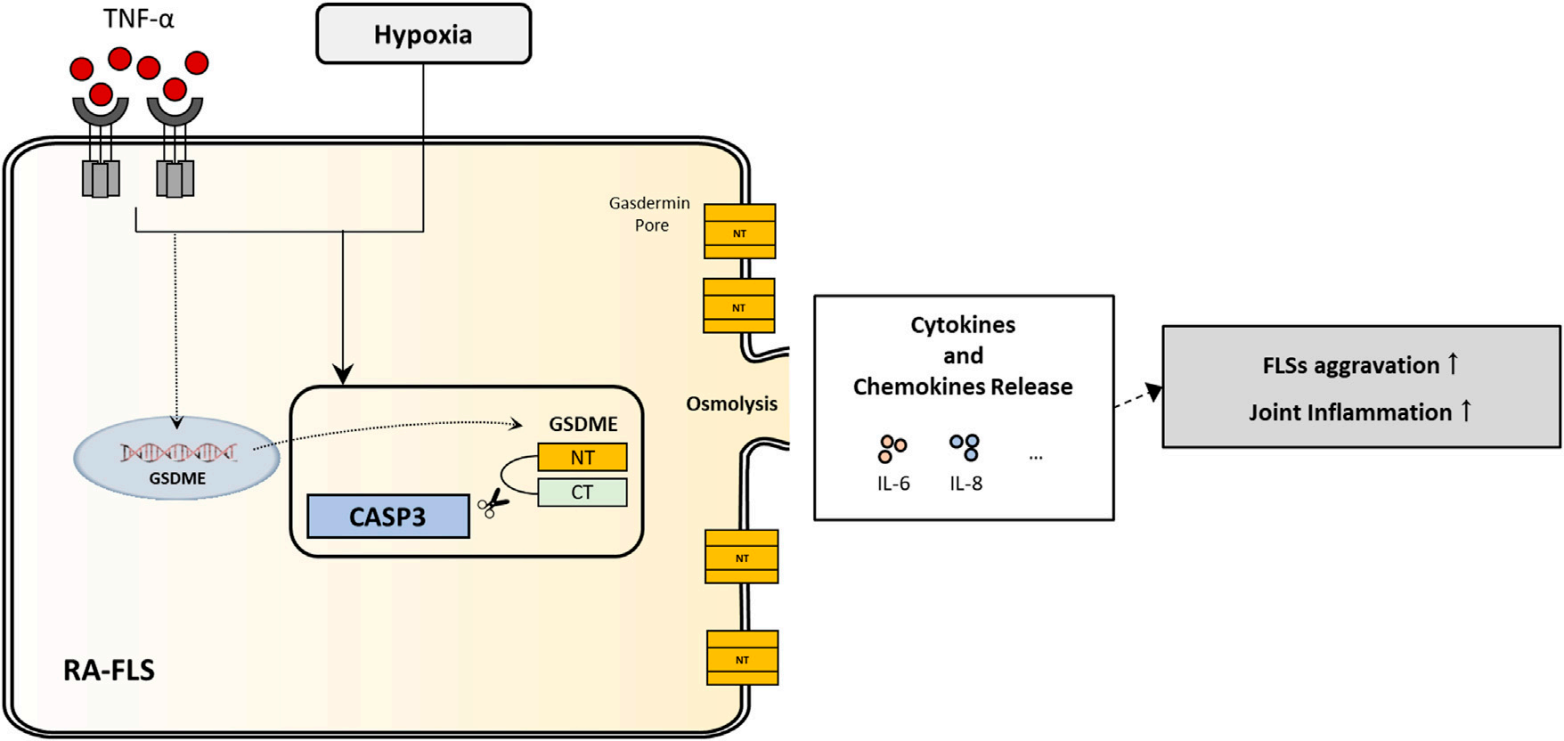
Illustration of a novel regulation of inflammatory cytokines secretion and aggressive cell behaviors in RA-FLSs through GSDME-mediated pyroptosis.

To study the roles of GSDME-mediated pyroptosis in RA-FLSs activation, we silenced GSDME expression with siRNA transfection and then determined the cell death rate, proliferation rate, migration and invasion ability, and inflammatory cytokines secretion of RA-FLSs. With GSDME suppression, the level of TNF-α+hypoxia-induced pyroptosis was significantly downregulated, along with restrained cell proliferation rate, migration/invasion ability and secretion of cytokines (especially IL-6). These results indicated that GSDME-mediated pyroptosis may contribute to the overactivation and aggressive behavior of RA-FLSs, inflammatory mediators leakage into the extracellular space and inflammatory cell recruitment from blood to synovium, which are critical mechanisms of cartilage and bone erosion and persistent synovitis (Figure 6).

The pathogenic role of IL-6 in RA has been widely demonstrated. IL-6 is an important regulator of B-cell maturation, T cell differentiation and bone homeostasis (McInnes and Schett, 2007; Garbers et al., 2018). IL-6 increases the expression of receptor activator of nuclear factor-κB ligand (RANKL), an important factor for osteoclast differentiation, by osteoblasts, disturbing osteoclast–osteoblast balance and exacerbating bone destruction (McInnes and Schett, 2007; Garbers et al., 2018). Fibroblasts are major contributors to synovial inflammation and bone/cartilage destruction and are dominant producers of a variety of cytokines, especially IL-6 in RA, FLSs-derived IL-6 also regulates the production of matrix enzymes, such as matrix metalloproteins (MMPs), aggrecanases and cathepsins, aggravating articular damage (McInnes and Schett, 2007; Nguyen et al., 2017). As a differentiation factor of TH17 cells, IL-6 is pivotal in local inflammation. Previous studies have shown that the high concentrations of IL-6 in synovial fluids positively correlate with disease activity and risk of radiographic progression in arthritis (McInnes and Schett, 2007; Larsson et al., 2015; Nguyen et al., 2017). The application of IL-6-targeted therapy, such as tocilizumab, significantly reduces IL-6 expression in RA synovium, demonstrating improvements in RA therapy (Kanbe et al., 2011; Kang et al., 2019). Nonetheless, understanding the mechanism of IL-6 (and probably other cytokines/chemokines) secretion by RA-FLSs through GSDME-mediated pyroptosis still provides a new perspective for developing more effective treatments in RA.

Regrettably, most experiments in this study were conducted in RA-FLSs cultured *in vitro*. Of note, the absolute level of pyroptosis induced by TNF-α+hypoxia remained less than 10%, indicating that only a small fraction of RA-FLSs underwent pyroptosis. However, a single spark can start a prairie fire. Recent studies reported a mechanism of cytokines release in the sublytic phase before the truly membrane-lytic pyroptosis phase (Xia et al., 2021; Zhou and Abbott, 2021), which might explain the significant effects resulting from pyroptosis of a small number of cells. Due to the limitation of direct detection methods of GSDME-NT *in situ* in synovial tissue, the actual pyroptosis level of RA-FLSs *in vivo* still requires further verification. A more reliable and accurate method to detect pyroptosis levels in tissue and larger scale of samples and patient cohort data are required for correlation analysis between GSDME-mediated FLSs pyroptosis and clinical parameters in the future. Another limitation of this research is the lack of animal study. But thanks to Zhai’s work, we can derive a key information from the CIA model of GSDME knockout mice that Gsdme^−/-^ mice exposed to collagen exhibited a reduced incidence of arthritis and lower clinical arthritis scores than wild-type mice, providing strong support of our results.

In conclusion, our study demonstrates a novel mechanism for modulating cell proliferation, migration, invasion and inflammatory cytokines secretion by GSDME mediated pyroptosis in RA-FLSs. These results provide new insight into the crosstalk between the inflammatory and hypoxic synovial microenvironment and the abnormally activated RA FLSs, thus suggesting a new direction for FLSs targeted therapy for RA.

## Data Availability

The original contributions presented in the study are included in the article/Supplementary Material, further inquiries can be directed to the corresponding author.
