# Supplementary material for: Gasdermin-E Mediated Pyroptosis—A Novel Mechanism Regulating Migration, Invasion and Release of Inflammatory Cytokines in Rheumatoid Arthritis Fibroblast-like Synoviocytes
Source: Front Cell Dev Biol. 2022 Feb 14;9:810635. doi: 10.3389/fcell.2021.810635 (PMC8882760; doi:10.3389/fcell.2021.810635)

**Supplementary Material**

**Supplementary Table**

**Table S1 Demographic and clinical characteristics of RA patients**

| Characteristics | RA (n=21) | OA (n=4) | Orth.A (n=5) | |
| --- | --- | --- | --- | --- |
| Age, yr., median (IQR) | 53 (40, 62) | 65 (55, 77) | 47 (36, 53) |  |
| Female, n (%) | 19 (86.4) | 2 (50.0) | 2 (40.0) |  |
| Disease duration, months, median (IQR) | 67 (24, 160) |  |  |  |
| TJC28, median (IQR) | 6 (3, 12) |  |  | |
| SJC28, median (IQR) | 4 (2, 7) |  |  |  |
| PtGA, median (IQR) | 5 (4, 8) |  |  |  |
| PrGA, median (IQR) | 5 (4, 6) |  |  |  |
| ESR (mm/h), median (IQR) | 58 (32 ,92) |  |  |  |
| CRP (mg/L), median (IQR) | 19.8 (6.3, 41.8) |  |  |  |
| Positive RF, *n* (%) | 17 (81.0) |  |  |  |
| Positive ACPA, *n* (%) | 19 (90.5) |  |  |  |
| DAS28-CRP, median (IQR) | 4.5 (3.7, 5.5) |  |  |  |
| CDAI, median (IQR) | 20 (15, 33) |  |  |  |

Abbreviations: RA, rheumatoid arthritis; SJC28, 28-joint swollen joint count; TJC28, 28-joint tender joint count; PtGA, patient global assessment of disease activity; PrGA, provider global assessment of disease activity; ESR, erythrocyte sedimentation rate; CRP, C-reactive protein; RF, rheumatoid factor; ACPA, anti-cyclic citrullinated peptide antibody; DAS28-CRP, disease activity score 28 with CRP; CDAI, Clinical Disease Activity Index.

**Supplementary Figures**

**Supplementary Figure 1 GSDME (DFNA5) gene signatures in the Pathobiology of Early Arthritis Cohort (PEAC).**

**(A)** Volcano plot view of gasdermin family genes in differentially expressed genes comparing RNA sequencing of synovial tissue. Lympho-myeloid (L), diffuse-myeloid (M), and pauci-immune fibroid (F) vectors are mapped to 3 axes using polar coordinates in the horizontal plane. **(B)** GSDME (DFNA5) gene expression level in different synovium pathotypes.  **(C)** Three axis polar plots of synovium modules derived by weighted correlation network analysis (WGCNA) in different pathotypes. **(D)** SC78 (THY1+) module view in different synovium pathotypes.  **(E)** Heatmap showing GSDME (DFNA5) expression in SC78 (THY1+) modules in synovium grouped by pathotype.

**Supplementary Figure 2 Immunofluorescent staining of RA (n = 3) and Orth.A (n = 3) synovial tissues with antibodies to GSDME (grey), CD55 (green) and THY-1 (red) or control IgG. All nuclei were counterstained with DAPI (scale bar = 100μm).**

**Supplementary Figure 3 Validation of GSDME knock-down efficiency**

**(A, B)** RA-FLSs are transfected with negative control siRNA (si-NC) or three different GSDME siRNA (si-GSDME#1/2/3). GSDME mRNA expression is evaluated by qPCR (**A**, n = 3) and protein expression is evaluated by western blot (**B**, n = 3). Data are shown as mean ± s.e.m and results are representative of three independent experiments. **P* < 0.05, ***P* < 0.01, ****P* < 0.001, *****P* < 0.0001.

**Supplementary Figure 4 Western blotting results showing GSDME-FL and GSDME-NT levels in synovium tissue extracts of RA (n = 26), OA (n = 4) and Orth.A (n = 3) patients.**


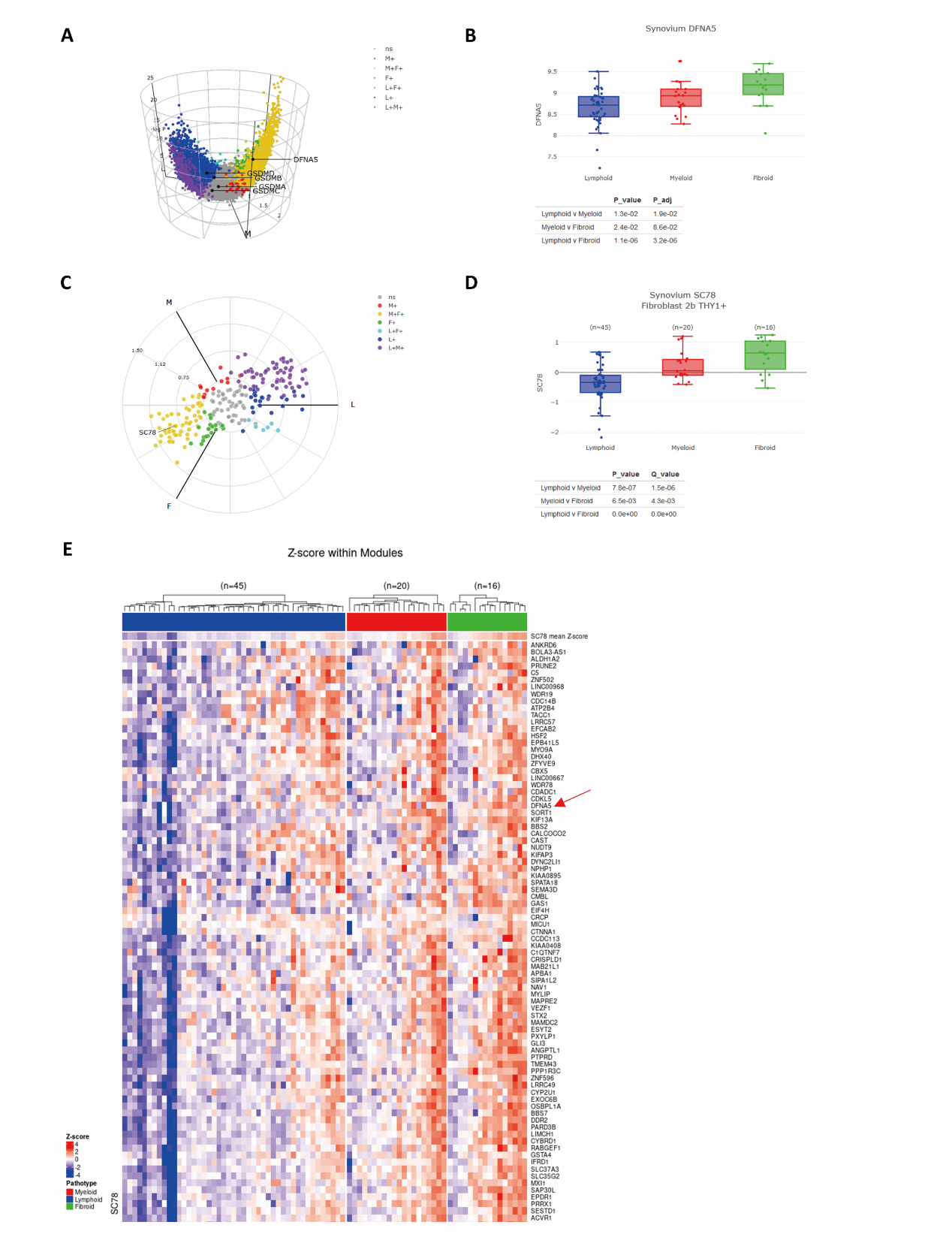


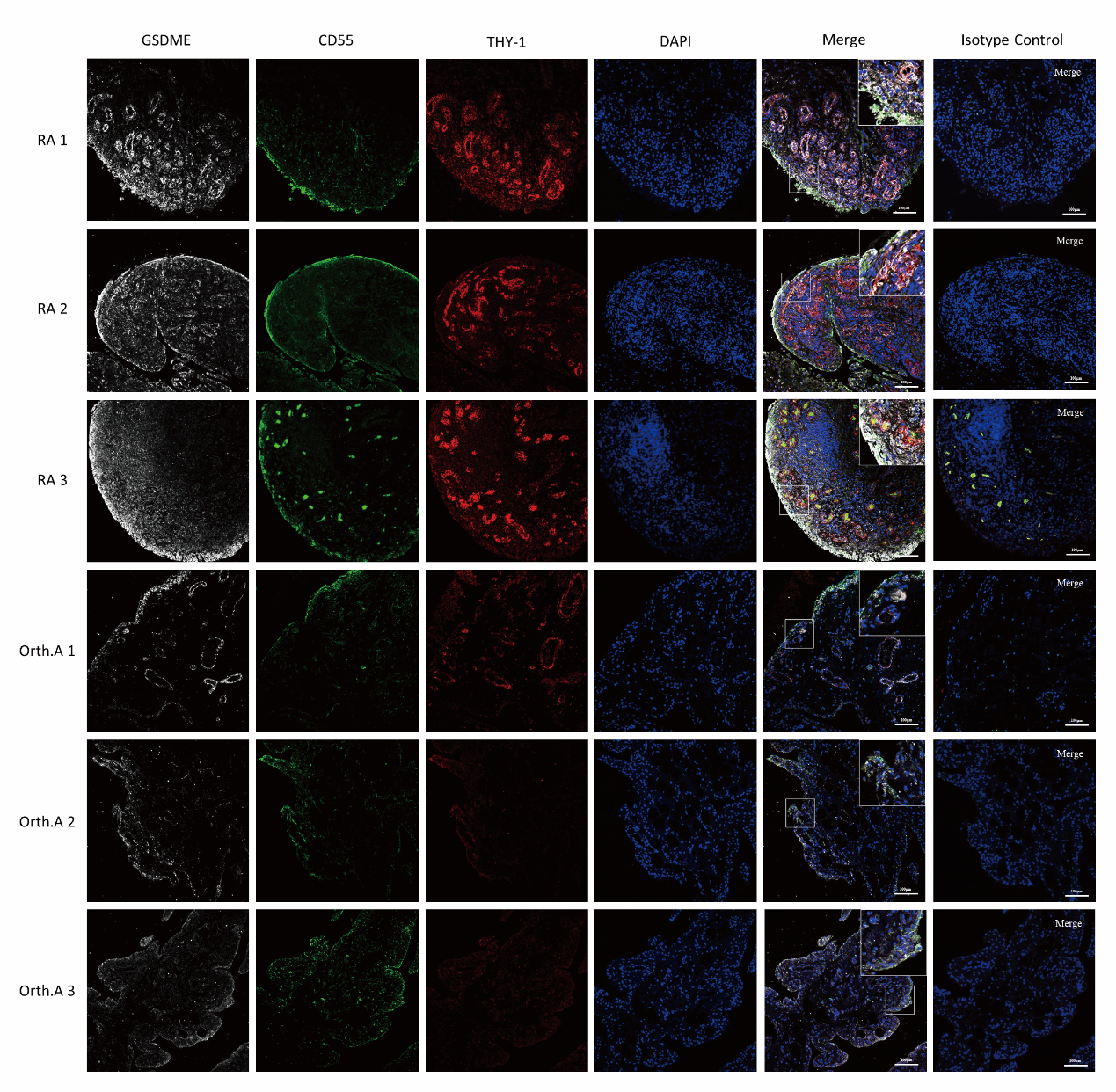


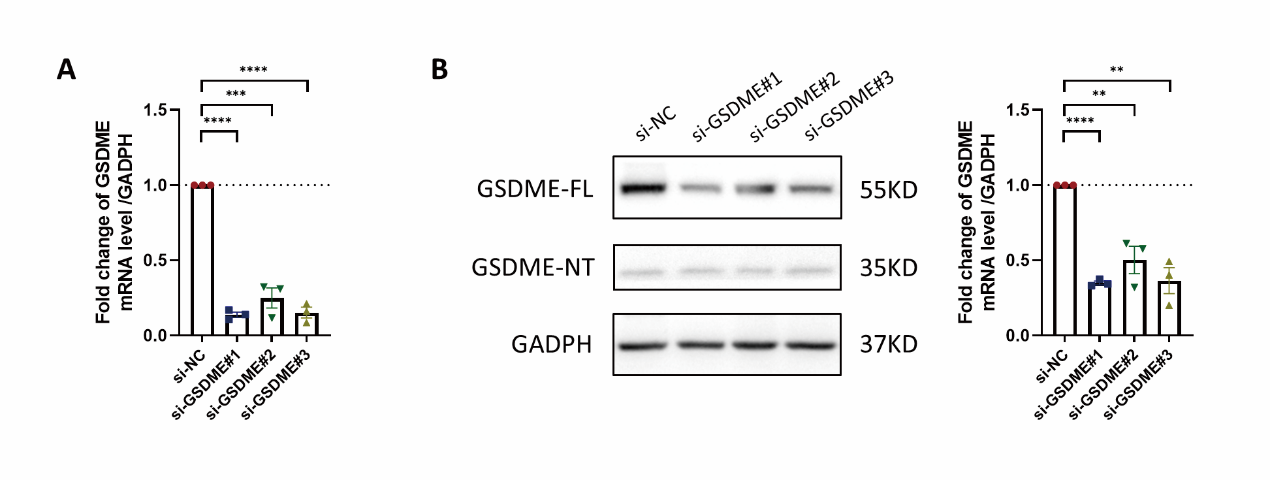


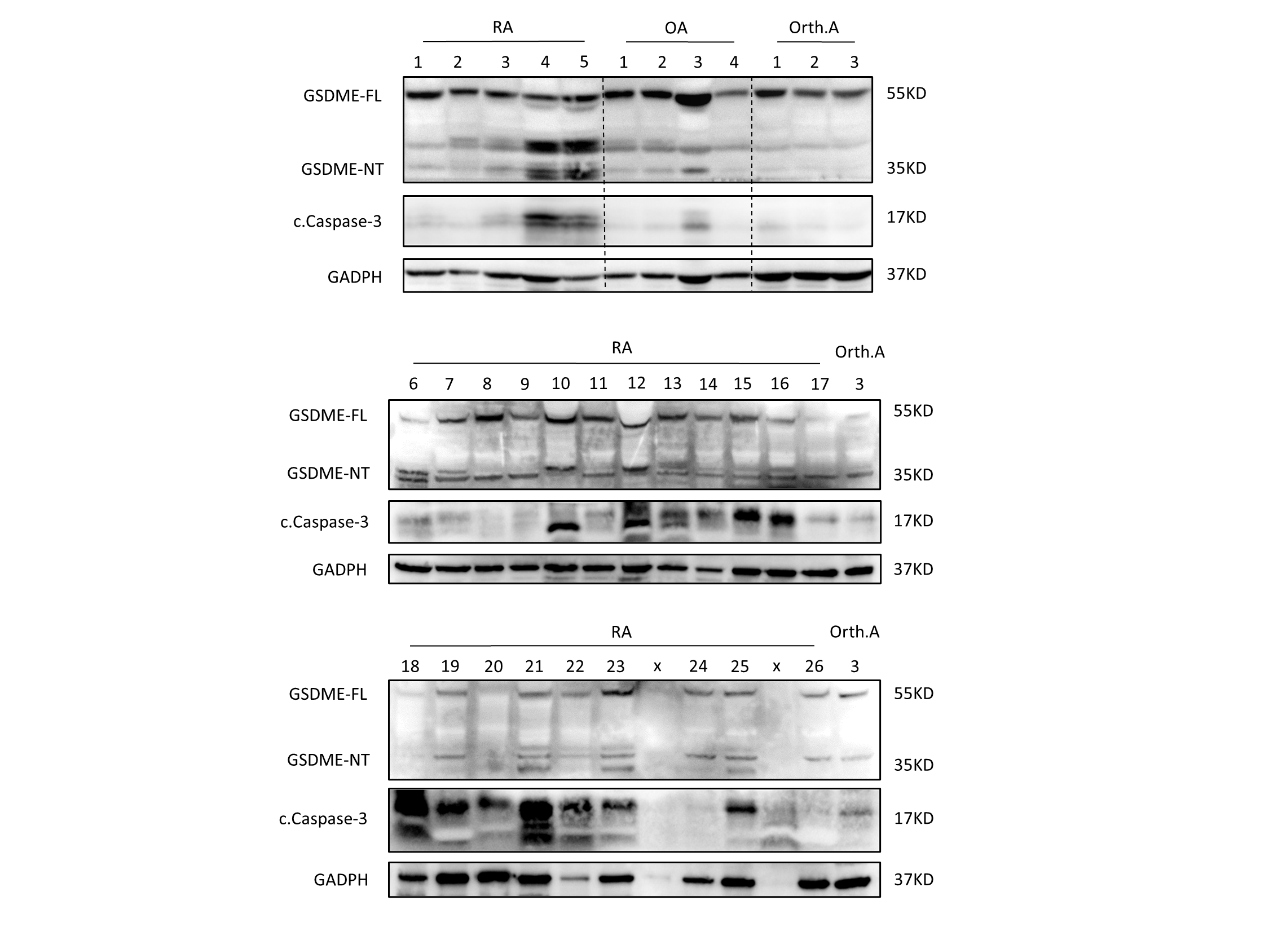

Supplement: Supplementary file 1 [file DataSheet1.docx]
